# NEUROENDOCRINE TUMOR IN A CHILD WITH COMMON VARIABLE IMMUNODEFICIENCY

**DOI:** 10.1590/1984-0462/2020/38/2018146

**Published:** 2019-11-25

**Authors:** Pedro de Souza Lucarelli Antunes, Heloísa Gabriel Tersariol, Mainã Marques Belém Veiga, Maria Conceição Santos de Menezes, Fabíola Del Carlo Bernardi, Wilma Carvalho Neves Forte

**Affiliations:** a School of Medical Sciences, Santa Casa de São Paulo, São Paulo, SP, Brazil.; b Irmandade da Santa Casa de Misericórdia de São Paulo, São Paulo, SP, Brazil.

**Keywords:** Child, Immunologic deficiency syndromes, Intestinal neoplasms, Criança, Síndromes de imunodeficiência, Neoplasias intestinais

## Abstract

**Objective:**

To report a case of a child with primary immunodeficiency who at eight years developed digestive symptoms, culminating with the diagnosis of a neuroendocrine tumor at ten years of age.

**Case description:**

One-year-old boy began to present recurrent pneumonias in different pulmonary lobes. At four years of age, an immunological investigation showed a decrease in IgG and IgA serum levels. After the exclusion of other causes of hypogammaglobinemia, he was diagnosed with a Common Variable Immunodeficiency and started to receive monthly replacement of human immunoglobulin. The patient evolved well, but at 8 years of age began with epigastrium pain and, at 10 years, chronic persistent diarrhea and weight loss. After investigation, a neuroendocrine tumor was diagnosed, which had a rapid progressive evolution to death.

**Comments:**

Medical literature has highlighted the presence of gastric tumors in adults with Common Variable Immunodeficiency, emphasizing the importance of early diagnosis and the investigation of digestive neoplasms. Up to now there is no description of neuroendocrine tumor in pediatric patients with Common Variable Immunodeficiency. We believe that the hypothesis of digestive neoplasm is important in children with Common Variable Immunodeficiency and with clinical manifestations similar to the case described here in the attempt to improve the prognosis for pediatric patients.

## RESUMO

**Objetivo:**

Relatar um caso de criança portadora de imunodeficiência primária que, aos oito anos, desenvolveu sintomas digestivos, culminando com o diagnóstico de tumor neuroendócrino aos dez anos de idade.

**Descrição do caso:**

Menino, com um ano de idade, começou a apresentar pneumonias de repetição em diferentes lobos pulmonares. Aos quatro anos, a investigação imunológica mostrou diminuição dos níveis séricos de IgG e IgA. Após exclusão de outras causas de hipogamaglobulinemia, teve diagnóstico de imunodeficiência comum variável, passando a receber reposição mensal de imunoglobulina humana. Evoluiu bem, porém, aos oito anos, começou com epigastralgia e, aos dez anos, diarreia crônica persistente e perda de peso. O quadro culminou com o diagnóstico de tumor neuroendócrino intestinal, de rápida progressão, com óbito do paciente.

**Comentários:**

A literatura tem chamado a atenção para tumores gástricos em adultos com imunodeficiência comum variável, alertando para a importância do diagnóstico precoce e da pesquisa de neoplasias digestivas. Até o momento, não há descrição de tumor neuroendócrino em pacientes pediátricos portadores de imunodeficiência comum variável. Acredita-se ser importante a hipótese de neoplasia digestiva diante de crianças com imunodeficiência comum variável e com manifestações clínicas semelhantes ao caso descrito, na tentativa de melhorar o prognóstico para pacientes pediátricos.

## INTRODUCTION

Common Variable Immunodeficiency (CVID) is a primary immunodeficiency disorder (PID) or an innate immunity error (IIE), which is a more common severe symptom, characterized by predominant antibody deficiency. Its incidence ranges from 1:50,000 to 1:200,000, and it is more common among Caucasians and rare among Asians and African descendants.^1^ ^,^ ^2^ Furthermore, it affects both genders equally.^3^

CVID is defined as decreased immunoglobulin G (IgG) and IgA, or IgM and specific antibody deficiency after ruling out other causes of hypogammaglobulinemia.^4^ For the class change from IgM to IgG and IgA, adhesion molecules expressed on T helper and B lymphocytes are needed, respectively: CD40L to CD40; Inducible Costimulatory molecule (ICOS) to ICOS-L; B-cell Activating Factor (BAFF) receptor to Transmembrane Activator and Calcium-modulator Ligand (TACI).^5^ For the final differentiation of B lymphocyte into plasmocyte, the presence of B differentiation groups is essential: CD19, CD20, CD21, CD81. ^5^ CVID has been linked to mutations, causing expression deficiencies of the following molecules: ICOS, BAFF, TACI, CD19, CD20, CD21 and CD81.^6^ The consequence is that B lymphocytes become unable to differentiate into plasmocytes, showing an abnormal response to immunization protocols against protein antigens and polysaccharides.^7^

As a result of antibody deficiency, 94% of CVID patients have recurrent infections, especially pneumonia, which may progress to chronic lung disease (29%) and bronchiectasis (11%). They may also manifest inflammatory bowel disease (15%), hematological or organ-specific autoimmune diseases and neoplasms.^7^ A subset of patients has dysfunctional cellular responses with opportunistic pathogen infections.^8^

Neoplasms and lymphoproliferative diseases occur more frequently in CVID patients compared to immunocompetent individuals and they have an incidence of lymphoma that is 300 times higher than in the general population.^9^ To date, there is no literature describing a case of neuroendocrine neoplasia in pediatric patients with CVID, which is why we have proposed to report the present case.

## CASE DESCRIPTION

A four-year-old boy from São Paulo, Brazil, was referred to a specialized unit for presenting recurrent pneumonia since he was one year old. The cases of pneumonia were in different lobes of the lungs, and there were often more than two occurrences a year, requiring several hospital admissions. At the initial time of care, the patient was active, eupneic, and had a weight at the lower limit of a normal range, with no other changes. There was a history of repeat infections in his cousin, who died as a teenager. The diagnostic hypothesis was PID, predominantly from antibodies. Tests performed for the immunological investigation showed: IgG 230 mg/dL (expected 739-1475); IgA <7 mg/dL (expected 113 to 248); IgM 124 mg/dL (expected 65-134); the lymphocyte subpopulations were: CD19+ 483 (460-1203), CD3+ 982 (1161-2077), CD4+ 302 (630-1182), CD8+ 661 (332-836) cells/mm^3^ ratio CD4/CD8=0.45 (expected ³1). Serology was negative for HIV, *Epstein-Barr virus* and cytomegalovirus. There were no intestinal or renal protein losses, no other diseases, no medication usage, and no exposure to specific pollutants.

Given the clinical and laboratory findings, a diagnosis of CVID was made and monthly intravenous human immunoglobulin replacement was introduced. The patient no longer had pneumonia, and there was rapid weight gain that reached a normal range. He was also taught how to maintain personal and environmental hygiene, avoiding the ingestion of raw foods outside his home.

He evolved well until he was eight years old when he began to have epigastric pain. Repeated parasitological stool tests were negative. An upper digestive endoscopy was recommended, and it showed a diaphragmatic hernia and a duodenal polyp, which was removed. A biopsy revealed tubular adenoma with (low grade) moderate dysplasia. Annual endoscopic follow-up was directed and symptomatic treatment started, with clinical improvement.

At the age of nine, another endoscopy was performed. It displayed images of reflux esophagitis and duodenal polyps, which were removed through endoscopy. Anatomopathological analysis revealed moderate chronic active esophagitis with eosinophilia, as well as duodenal tubular adenomas with intense (high-grade) cytological changes and moderate chronic duodenitis. On the same occasion, a colonoscopy was performed, which showed a low-grade tubular-adenoma with moderate and mild atypia in the colon mucosa and a low-grade tubular adenoma in the rectal mucosa. All adenomas were removed endoscopically.

At the age of ten, the patient presented pneumonia on two occasions, which required prolonged hospitalization. During this period, he began to have severe diarrhea, which became chronic. The parasitological stools remained negative. He evolved with a weight loss of 15 kg. He was 125 cm tall (percentile for weight and height <3), and had electrolyte disturbance. New endoscopies were attempted several times, but the patient’s clinical condition contraindicated the examination. Thus, an abdominal computed tomography was opted for, which showed extensive intestinal pneumatosis, diffusely affecting the rectum, sigmoid, descending colon and transverse colon. Fasting was prescribed and a parenteral diet was introduced. The condition improved and a hypercaloric enteral diet was started.

**Figure 1:**
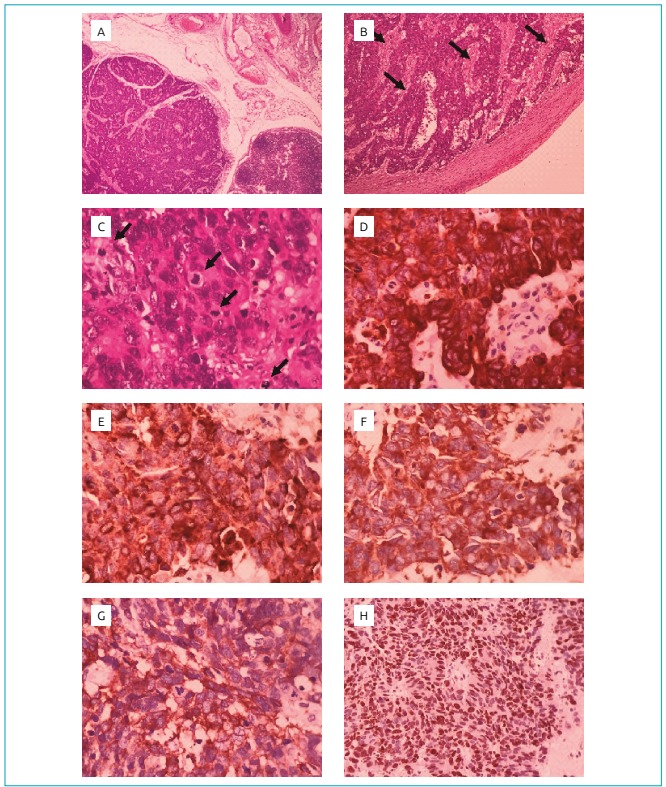
(A) Infiltrating/metastatic neuroendocrine carcinoma characterized by the proliferation of small and intermediate cells with anaplasia, arranged in blocks and cords (Hematoxylin-Eosin, 40x); (B) the presence of necrosis areas (arrows) between neoplastic cell blocks and cords (Hematoxylin-Eosin, 100x); (C) the presence of frequent figures of atypical mitoses (arrows) (Hematoxylin-Eosin, 400x); (D) immunohistochemical expression of pancytokeratin (AE1-AE3, 400x); (E), (F), (G) immunohistochemical expression of neuroendocrine markers (Chromogranin A, Synaptophysin and CD56, 400x, respectively); (H) high proliferation rate to KI67, positive in 80% of neoplastic cells (KI67, 200x).

He presented clinical improvement and gradual weight gain in the following four months, when the diarrhea ceased. However, a month later, he had abdominal pain and rapidly progressing constipation. A new colonoscopy was performed, which showed a colon tumor mass that caused intestinal obstruction. Surgery was performed to clear the intestinal transit associated with the colostomy. Surgical removal of the tumor was not possible due to its large size. During surgery, metastases were also observed in the liver, omentum and costal mesh. The anatomopathological result of the intestinal tumor and metastasis was neuroendocrine neoplasia with a high cell proliferation index. Immunohistochemical research revealed the immunoexpression of chromogranin, synaptophysin, KI67 (which was positive in 80% of the cells). After the diagnosis, two cycles of chemotherapy were performed, but the patient died two months later (Figure 1).

## DISCUSSION

The patient had some criteria that suggest PID, according to the warning signs indicated by the Brazilian Immunodeficiency Group (*Grupo Brasileiro de Imunodeficiências* - BRAGID): two or more episodes of pneumonia in the last year; eight or more ear infections in the last year; recurrent stomatitis or persistent moniliasis for more than two months; recurrent abscesses or ecthemya; severe systemic infections (meningitis, osteoarthritis or septicemia); recurrent bowel infections or chronic diarrhea; severe asthma, collagen disease or autoimmune disease; adverse effects on BCG and/or mycobacterial infection; clinical phenotype suggestive of immunodeficiency associated syndromes; family history of immunodeficiency.

Repeated episodes of pneumonia, a family history of death from infections and complementary exams with normal B lymphocytes and deficiency of two classes of immunoglobulin (IgG and IgA) led to the diagnostic hypothesis of CVID. In addition, other causes of hypogammaglobulinemia were ruled out in order to diagnosis CVID: congenital agammaglobulinemia (normal CD19), hyper-IgM syndrome (normal IgM and no other manifestations), Good syndrome (normal CD19 and CD3), X-linked lymphoproliferative syndrome (seronegative for *Epstein-Barr virus*), no intestinal or renal protein loss, no leukemia or lymphoproliferative disease, and no medication usage.

In CVID, there is recurrent pneumonia resulting from decreased IgG2 class anti-polysaccharide antibodies, which are needed to defend against encapsulated bacteria such as *Streptococcus pneumoniae* and *Haemophilus influenzae*.^5^ The replacement with human immunoglobulin allowed for the defense against such agents, and the patient no longer had pneumonia, as described in the literature.^4^

The patient also had a laboratory picture of IgA deficiency, which is part of CVID. The lack of IgA can lead to tonsillitis, otitis, recurrent diarrhea and giardiasis, which were not presented by the patient in question. Most cases of selective IgA deficiency are asymptomatic. Even so, the patient was directed to strengthen their personal and environmental hygiene in order to prevent mucosal infections that would require IgA.^5^ Diarrhea appeared only at the age of ten. It was chronic, uncontrollable, and led to malnutrition,with negative tests for infectious agents. All of these facts demonstrate that diarrhea was not a consequence of IgA deficiency.

Digestive manifestations at the age of eight, initially with epigastralgia and later with chronic diarrhea and weight loss, culminated in a diagnosis of neuroendocrine intestinal tumor, a neoplasm that has not yet been described in pediatric patients with CVID. Neuroendocrine neoplasms most commonly affect the gastrointestinal tract, especially the small intestine (1.08/100,000); the colon (0.40/100,000) and the rectum (1.05/100,000); and the pancreas (0.43/100,000).^10^ They present slow growth and indolent evolution.^10^ However, in the present case, the neuroendocrine tumor rapidly evolved into a large tumor mass and metastases.

Neoplasms, in general, have been described after ten years of evolution from the onset of clinical manifestations of CVID and, therefore, mainly in adults.^11^ The association between CVID and risk of gastric neoplasms in adults is described, with a 50 times higher risk for CVID compared to the average risk of the population. Regarding the development of gastric tumors in CVID, the frequent malignancy of this neoplasia is associated with factors such as pernicious anemia, gastric atrophy, achlorhydria and chronic infections from *Helicobacter pylori.* ^7^ ^,^ ^10^ ^,^ ^12^ In addition to gastric tumors in CVID, neoplasms of lymphohematopoietic and genital origin are reported, induced by persistent oncogenic virus infections. ^13^

The increased frequency of malignant neoplasms in CIVD patients may be associated with numerous descriptions of tumor suppressor gene changes, such as p53 point mutation, not only in patients with gastric cancer but also in precancerous lesions.^13^ Another factor is related to NK cells. Research does not show decreased NK82 cell numbers or NK56 cytotoxic activity in PIDs.^14^ However, NK cells are known to express a CD16 or FcγRIII surface receptor, which binds to the Fc portion of the IgG1 and IgG3 subclasses, mediating the phenomenon of antibody-dependent cellular cytotoxicity (ADCC), which is important in the response against neoplastic and virus-infected cells.^5^ In CVID, the decrease in IgG could lead to lower NK cell-mediated ADCC, compromising defense against neoplastic cells. On the other hand, it is still suggested that in CVID, the cytotoxic activity of NK cells is decreased even in the presence or absence of IL-2, pointing to a defect in lymphokine-activate-killer (LAK) cell activity.^14^ It is therefore possible that the different hypotheses described lead to the rapid evolution of malignancies in patients with CVID.

The present report makes us conclude that the diagnosis of neuroendocrine intestinal tumor should be made not only in adults, as described in the literature, but also in pediatric patients with CVID. Thus, the hypothesis of digestive tumors should be considered in patients with CVID who have epigastric pain, chronic diarrhea of an unknown cause and weight loss. It is likely that cases of digestive adenomas in patients with CVID should be further investigated in an attempt to diagnose aggressive neoplasms. It is also possible that if the patient had been diagnosed earlier with a neuroendocrine tumor, his neoplasia could have been more successfully treated and removed. We believe that this report may alert health professionals to the possibility of neuroendocrine tumors in pediatric patients with CVID, in an attempt to provide better clinical evolution for these children

## References

[1] Cunningham-Rundles C, Bodian C (1999). Common variable immunodeficiency: clinical and immunological features of 248 patients. Clin Immunol.

[2] Oksenhendler E, Gérard L, Fieschi C, Malphettes M, Mouillot G, Jaussaud R (2008). Infections in 252 patients with common variable immunodeficiency. Clin Infect Dis.

[3] Bonilla FA, Barlan H, Costa-Carvalho BT, Cunningham-Rundles C, Morena MT, Espinosa-Rosales FJ (2016). International Consensus Document (ICON): Commom variable immunodeficiency disorders. J Allergy Clin Immunol Pract.

[4] Goudouris ES, Rego Silva AM, Ouricuri AL, Grumach AS, Condino-Neto A, Costa-Carvalho BT (2017). II Brazilian Consensus on the use of human immunoglobulin in patients with primary immunodeficiencies. Einstein (Sao Paulo).

[5] Forte WCN, Forte WC (2015). Imunodeficiências primárias. Imunologia do básico ao aplicado.

[6] Yong PF, Tarzi M, Chua I, Grimbacher B, Chee R (2008). Common variable immunodeficiency: an update on etiology and management. Immunol Allergy Clin North Am.

[7] Resnick ES, Moshier EL, Godbold JH, Cunningham-Rundles C (2012). Morbidity and mortality in common variable immune deficiency over 4 decades. Blood.

[8] Kokron CM, Errante PR, Barros MT, Baracho GV, Camargo MM, Kalil J (2004). Clinical and laboratory aspects of common variable immunodeficiency. An Acad Bras Cienc.

[9] Errante PR, Condino-Neto A (2008). Common variable immunodeficiency: a comprehensive review. Rev Bras Alerg Imunopatol.

[10] Fraenkel M, Kim M, Faggiano A, de Herder WW, Valk GD (2014). Knowledge NETwork. Incidence of gastroenteropancreatic neuroendocrine tumours: a systematic review of the literature. Endocr Relat Cancer.

[11] WHO Scientific Group (1995). Primary Immunodeficiency Diseases. Report of a WHO Scientific Group. Clin Exp Immunol.

[12] Dhalla F, da Silva SP, Lucas M, Travis S, Chapel H (2011). Review of gastric cancer risk factors in patients with common variable immunodeficiency disorders, resulting in a proposal for a surveillance programme. Clin Exp Immunol.

[13] Graudenz GS, Oliveira CH, Pinho AJ, Lazzarini S (1999). Severe Complications in Common Variable Immunodeficiency: two case reports. Rev Bras Alerg Imunopatol.

[14] Eckert K, Schmitt M, Garbin F, Wahn U, Maurer HR (1994). Thymosin alpha 1 effects, in vitro, on lymphokine-activated killer cells from patients with primary immunodeficiencies: preliminary results. Int J Immunopharmacol.

[15] Ferriani VPL, Barbosa JE, Carvalho IF (1999). Complement haemolytic activity (classical and alternative pathways), C3, C4 and factor B titres in healthy children. Acta Paediatr.

